# Molecular Determinants of Seminal Plasma on Sperm Biology and Fertility

**DOI:** 10.3390/ijms22073555

**Published:** 2021-03-30

**Authors:** Manuel Álvarez-Rodríguez, Felipe Martinez-Pastor

**Affiliations:** 1 Department of Biomedical & Clinical Sciences (BKV), BKH/Obstetrics & Gynaecology, Faculty of Medicine and Health Sciences, Linköping University, SE-58185 Linköping, Sweden; 2 Department of Animal Health and Anatomy, Veterinary Faculty, Universitat Autònoma de Barcelona, 08193 Barcelona, Spain; 3 INDEGSAL, Department of Molecular Biology (Cell Biology), Universidad de León, 24071 León, Spain

Seminal plasma has gained attention in the last decades, developing from being a mere vehicle for spermatozoa delivery to the female to having a pivotal role in fertility and offspring well-being. Research done in recent years has offered us a wealth of information on the molecular composition of this biological fluid and its influence on sperm biology, fertilization, and pregnancy. Seminal plasma components modulate sperm physiology and interact with the female genital tract at many levels, affecting sperm transport and the immunological environment. Frontier research areas, such as proteomics, mRNA, miRNA, and extracellular vesicles analysis, have entered the study of seminal plasma. Moreover, differences between species are plentiful, reflecting the effect of strong evolutionary forces on an immense diversity of mating systems, genital morphology, and molecular interactions. Thus, seminal plasma has unique features and roles in each species, motivating a thriving research field focused on unraveling the purpose, composition, and functions of this fascinating fluid. Consequently, applied research on seminal plasma has also been abundant. Both friend and foe, techniques related to assisted reproduction have aimed either at suppressing adverse effects of seminal plasma or using it to improve fertility results. Consequently, research groups are currently dedicated to using seminal plasma or isolated components as supplements for sperm conservation, artificial insemination, or to promote embryo production or implantation.

This special issue of the International Journal of Molecular Sciences dedicated to the molecular determinants of seminal plasma on sperm biology and fertility has successfully published 17 outstanding papers, four reviews, and thirteen research articles. These publications consider many of the most significant areas in this field, and both the specialist and the occasional reader will find a complete view both on basic and applied aspects of seminal plasma.

The effect of vasectomy and the photoperiodic regime was studied in ram semen [1], finding that vasectomy modifies the protein profile and hormonal content of ram seminal plasma. In contrast, exposure to a constant photoperiod affects hormonal concentration and antioxidant enzymes activity. These molecular findings are of particular interest for small ruminants breeding, especially for those breeds subjected to higher seasonal variability. They also open avenues on how the environment affects seminal plasma composition and effects.

In boar, Pavaneli et al. present evidence that the presence of seminal plasma during liquid storage modulate in vitro capacitation and acrosomal exocytosis [2], shedding light on some events of relevance for pig breeding. Following this line, three candidate proteins (the granulin precursor, legumain, and AWN) could indicate SP-tolerance of sperm during long-term storage when using autologous seminal plasma in the extender [3]. Continuing with seminal plasma proteins effects and processing, Tumova et al. have demonstrated that the Ubiquitin-Proteasome system is not involved in the degradation of porcine β-Microseminoprotein during sperm capacitation [4], suggesting that the capacitation-induced processing of seminal plasma proteins on the sperm surface may be more complex than previously thought. The interaction of sperm with the different reproductive environments is another fascinating field of research. Luongo et al. show how seminal plasma and the uterine and oviductal fluids alter the sperm proteome [5], paving for further studies to understand sperm transport, selection, capacitation, and fertilization. Nevertheless, the effect of seminal plasma is not limited to the initial steps of reproduction, as stated by Martinez et al. [6]. These authors demonstrate that seminal plasma infusion before AI upregulates the expression of embryo development-related genes in Day 6 pig embryos.

In humans, Fraczek et al. evaluate genital heat stress through oxidative stress analysis [7], highlighting the relevance of this analysis in the context of novel treatment algorithms (targeted therapies). Regarding seminal plasma proteomics analysis for new fertility biomarkers, Fernández-Encinas et al. [8] show an association of ubiquitin C with sperm DNA fragmentation. This reveals that seminal plasma components could play a vital regulation role in the integrity of the sperm genetic material. Besides, a plausible marker of male subfertility was proposed, the seminal plasma miR-34b-5p microRNA [9], correlated to low sperm concentration. Semenogelin 1 (SEMG1), present human seminal plasma and involved in sperm motility and fertility regulation, were studied in mice [10], suggesting potential clinical applications for effective internal fertilization.

The study of fractionated seminal plasma protein use in stallion [11] did not significantly affect the sperm plasma membrane integrity and capacitation status. Nevertheless, seminal plasma proteins’ effect on sperm functionality is concentration-dependent, as has been reported for other species. In donkey, the activation of polymorphonuclear neutrophils is triggered by semen, suggesting a crucial role in the reproductive strategy of this species [12].

Finally, the microRNA analysis in salmon (*Salmo salar*) [13] indicates a high presence of these non-coding molecules on seminal plasma. Moreover, testicular-supporting somatic cells would be the most likely source.

Besides, four reviews were published [14,15,16,17], including an up-to-date revision of human seminal plasma factors that affect fertility both at functional and mechanistic level [14] and also the application of transcriptomics and proteomics in elucidating the molecular markers of idiopathic infertility [15]. One increasingly interesting topic—exosomes—is also covered to address the necessity of novel diagnostics tools on male infertility [16]. Finally, the study of divergent models, such as avian species, highlights the molecular basis of the seminal plasma’s effect in exogenous procedures as cooling and freezing, largely used in genomic resources banking [17].
